# A desire for authoritative science? How citizens’ informational needs and epistemic beliefs shaped their views of science, news, and policymaking in the COVID-19 pandemic

**DOI:** 10.1177/09636625211005334

**Published:** 2021-04-10

**Authors:** Senja Post, Nils Bienzeisler, Mareike Lohöfener

**Affiliations:** University of Göttingen, Germany

**Keywords:** interaction experts/publics, media and science, media representations, policy and science, risk communication, science communication, science in democracy, science journalism

## Abstract

The coronavirus pandemic created a situation in which virological and epidemiological science became highly politically relevant but was uncertain and fragmented. This raises the question as to how science could inform policymaking and public debate on societal crisis management. Based on an online survey of Germans (*N* = 1513) representative for age, gender, education, and place of residence, we investigate citizens’ prescriptive views of the relationships between science, policymaking, and the media. Views differ depending on their informational needs and epistemic beliefs. People with a need for definite information and a view of scientific knowledge as static wanted scientists to dominate policymaking and journalists to deliver definite information about the coronavirus. People with an informational need to construct their own opinions wanted journalists to question policy and scientific advice. Furthermore, they rejected the idea of scientists dominating policymaking. Results are discussed with reference to theories of science and democracy.

In many countries, as the gravity of the COVID-19 pandemic became clear, the demand of scientific expertise increased heavily among policymakers, journalists, and their audiences. Yet, the relationship between scientists, policymakers, and the public was not free of conflict. Audiences could observe an instance of this in a talk show hosted on the German public TV channel ARD in mid-March 2020, just after the so-called lockdown had become effective, including contact bans and closures of shops, schools, theaters, restaurants, and other public spaces. When a guest criticized that politicians had been too hesitant to close schools, North Rhine Westphalia’s Prime Minister Armin Laschet passed the accusation on with a sarcastic undertone stating that bright virologists had told him that it would not help to close schools.^1^ This prompted another guest of the talk show, Alexander Kekulé, a physician and expert of pandemics, to reject the criticism, pointing out that scientists could not be held accountable for political decision making.

Theorists of knowledge, science, and democracy have repeatedly argued that in science-based policymaking, the distinction between science and policy must be clear-cut to enable citizens to acknowledge scientific facts while engaging in political disputes over values and societal priorities (e.g. Pielke, 2004; Popper, 1957; Weber, 1904). Empirically, however, it appears that some members of the public hold strong preferences for delegating political decision making to scientists or expert circles, for example, for the sake of efficiency or because they cannot cope with ambivalence in public discourse (e.g. Hibbing and Theiss-Morse, 2003). An instance of this could be observed a few weeks later in a radio interview, when Armin Laschet declared that virologists would not tell him what decisions he had to make.^2^ Cited widely in the media, this statement drew extensive criticism, for example, among social media users accusing Laschet of ignoring science.^3^

The apparent odds that can exist between citizens’ and theoretical thinkers’ views of the relationship between science, policy, and the public debate warrant empirical research. We present the results of a study conducted in the context of the early phase of the coronavirus pandemic in Germany—at a point in the first so-called lockdown just before individual public figures started to discuss strategies to loosen the contact bans. We investigate how people’s normative views of the relationships between policymakers, scientists, and journalists relate to their approval of the public pandemic crisis communication, their informational needs, and their beliefs about the nature of scientific knowledge.

## 1. Science and policy

For science, the coronavirus pandemic created a situation that researchers have termed “post-normal” contrasting it with Thomas Kuhn’s (1970) classic concept of “normal science” (Funtowicz and Ravetz, 1994). Normal science denotes a long-term endeavor in which scientists engage to enlarge and refine existing scientific knowledge. Largely independent of society, they continuously derive hypotheses from theories produced by former research. In this process, scientists typically identify and solve isolated, increasingly specific research problems (“puzzles”), while society’s “really pressing problems, e.g. a cure for cancer or the design of a lasting peace, are often not puzzles at all, largely because they may not have any solution” (Kuhn, 1970: 36–37).

“Post-normal science” arises in urgent social crises, when available scientific knowledge is highly uncertain and incomplete while the stakes are high and values are disputed (Brüggemann et al., 2020; Funtowicz and Ravetz, 1994). The coronavirus pandemic gave rise to such a situation when secured virological and epidemiological knowledge was scarce, human losses and socioeconomic costs were at stake, and value conflicts had to be solved, for example, regarding individuals’ personal liberties and the protection of the vulnerable. Under these circumstances, the production of scientific knowledge was strongly oriented toward the political necessities of managing the crisis—giving rise to the question as to how scientific knowledge can translate into political decision making.

The relationship between science and policymaking has been controversially discussed in academia (e.g. Nelson and Vucetich, 2009; Pregernig, 2014). In various public conflicts over the environment, science, and technology, philosophers and sociologists of science have observed a naïve, that is, “linear,” view of the link between scientific knowledge and political decision making among members of the public, political decision makers, and scientists (Grundmann and Rödder, 2019; Pielke, 2004; Sarewitz, 2004). According to this view, political action can be derived from scientific knowledge in an unambiguous way, and scientific knowledge can make particular policy programs compelling (Grundmann and Rödder, 2019; Pielke, 2004).

Social scientists and philosophers of science have been largely critical of this view arguing that, even if based on scientific knowledge, political decision making necessarily involves value judgments—for example, when prioritizing desired political outcomes or weighing the benefits and costs of certain policy measures (Pielke, 2004; Sarewitz, 2004; Weber, 1904). In this vein, the function of science in policy debates is not to prescribe but to inform political decision making by identifying and comparing specific policy options against one another in a systematic way (Grundmann and Rödder, 2019; Pielke, 2004; Weber, 1904).

Scholars agree that, to achieve this, scientists and policymakers need to be aware of and transparent about the boundaries between science and policy, that is, about the line “where the thinking scientist stops and the desiring man starts speaking” (Weber, 1904: 33; see also Pielke, 2004).^4^ However, in public debates involving post-normal science, some scientists and policymakers have been found to blur this line using scientific knowledge as seemingly definite arguments for or against particular lines of political action (Lupia, 2013; Pielke, 2004; Pielke and Sarewitz, 2002; Post and Ramirez, 2018; Pullin et al., 2009; Sarewitz, 2004; Scheufele, 2014). What is more, members of the general public appreciate scientists’ political involvement. For instance, many favor a delegation of political decision making to expert circles because they prefer efficiency over disagreement or because they believe that scientists can best decide on what is good for society (Brossard and Shanahan, 2003; Hibbing and Theiss-Morse, 2003; Howell et al., 2020).

To conclude, several theories of knowledge and democracy put forth that scientists and policymakers should be clear about the boundaries of their specific domains of reasoning to enable citizens to engage in disputes over values and priorities (Pielke, 2004; Weber, 1904). The degree to which this is feasible depends, among others, on citizens’ conceptualizations of the relationship between science, policy, and the public debate. We investigate this issue in the context of the early phase of the coronavirus pandemic in Germany.

## 2. Science and the media

Most people learn about policy relevant scientific issues via the mass media (e.g. Metag et al., 2018). This raises the question as to how media cover policy relevant science in general and how they covered the pandemic in particular. For the media, the coronavirus pandemic created a highly undetermined situation—a complex development rapidly evolving into an existential crisis with a highly uncertain factual base. In such situations, compensating for uncertainty, journalists typically make their news judgments on “what is true (facts), relevant (agenda) and acceptable (opinion)” through social validation in their professional peer group (Donsbach, 2004: 140). This can lead to convergent news judgments just when states of affairs are highly contingent, for instance, because of knowledge gaps or because they involve a plurality of views from different angles (e.g. Fishman, 1978; Reinemann, 2004; Zelizer, 1993). Journalists’ striving for certainty and definite facts often leads to discrepancies between media representations of science and scientific knowledge. Scientific knowledge inherently features uncertainty, ambiguity, and knowledge gaps, which journalists typically seek to avoid (Dunwoody, 1999; Post, 2013, 2016; Stocking and Holstein, 1993). The erroneous impression of tentative scientific knowledge as certain is often perpetuated in mediated public debates when the media cite politically interested actors exploiting pieces of knowledge as seemingly definite arguments for or against particular lines of policy (Kepplinger et al., 1991; Pielke, 2004).

As the pandemic and its societal management involved highly complex and specific scientific knowledge, the demand for science journalism grew instantly. At the beginning of the crisis, Germany’s top virologist Christian Drosten was cited widely stating that science journalism had obtained a vital societal function.^5^ For years in many countries, however, the financial resources of science journalism have declined sharply. At the same time, financial resources of academic science public relations (PR) grew markedly (cf. Schäfer, 2017). As science PR has become gradually more professionalized and adapted to the media’s needs, journalists have increasingly accepted science press releases as a welcome resource often publishing them without further editing (Autzen, 2014; Brechman et al., 2011; Sumner et al., 2014; Vogler and Schäfer, 2020).

These general findings on journalists’ crisis and science news coverage resonated with media criticism during the early phase of the coronavirus pandemic in Germany. For the first months of the crisis during the so-called lockdown, several media scholars diagnosed a highly convergent and uncontested view of the pandemic with a concentration on few scientific sources (Boberg et al., 2020; Jarren, 2020; Wormer, 2020). Media scholars also criticized that many journalists merely announced scientists’, government experts’, and policymakers’ empirical data, statistical estimates, judgments, and conclusions—rather than putting them in context or questioning them (cf. Wormer, 2020).^6^ In accordance with this criticism, the German media appear to have compensated the prevailing uncertainty by highly convergent and largely undisputed news coverage in the early phase of the pandemic. It is an open question, however, to what degree the academic criticism resonated with news audiences’ views and expectations. We will turn to this question in the following.

## 3. Audience expectations and evaluations

How did citizens conceptualize the relationship between scientists, policymakers, and the public in the coronavirus pandemic, and how content were they with the public pandemic crisis communication? It is plausible that answers to these questions relate to citizens’ informational needs and views of the nature of scientific knowledge (cf. Bromme et al., 2010).

### Informational needs

For citizens, the coronavirus pandemic created a major social crisis potentially affecting individuals’ personal health, and psychological and economic well-being with a highly uncertain outcome. In this situation, people had a strong need for information resulting in a surge of news consumption in Germany and elsewhere (Nielsen et al., 2020; Peter and Brosius, 2020). Chew (1994) differentiates between an *orientational* and a *constructive* informational need. An informational need for orientation represents a desire for definite information potentially reducing knowledge gaps. It relates to questions, such as “What are the specifics of the situation?” or “What do the experts say?” By contrast, a need for construction represents a desire for different views to make up one’s own mind. It relates to questions, such as “What are the different viewpoints so that I can select the best position?” or “What information helps me develop an opinion?”

Findings on citizens’ views of democratic decision making suggest that individuals’ informational needs relate to their normative views of the cooperation between scientists and policymakers in times of crisis. Researchers have investigated citizens’ *deference to scientific authority*, that is, “the extent to which people believe that decision-making concerning science and technology should be the purview of the scientific community and not part of larger democratic discourse” (Howell et al., 2020: 1). Among others, deference to scientific authority is high among dogmatic, close-minded individuals who tend to disregard opposing views (Howell, 2019). These findings resonate with findings on citizens’ support of “stealth democracy,” that is, a preference among citizens to preclude public debate and to delegate political decision making to expert circles for the sake of efficiency (Hibbing and Theiss-Morse, 2003). The support of stealth democracy is especially high among individuals with an aversion for disagreement and conflict (Hibbing and Theiss-Morse, 2003). In view of these findings, we assume that people’s informational preferences in the pandemic are associated with their normative expectations of the relationship between scientists and policymakers, with those seeking definite information for orientation favoring, and those seeking information for constructing their own opinions criticizing dominance of scientists in policymaking. We hypothesize the following:

**Hypothesis 1a (H1a):** The more individuals sought information for orientation in the pandemic, the more they wanted scientists to dominate pandemic policymaking.
**Hypothesis 1b (H1b):** The more individuals sought information for construction, the less they wanted scientists to dominate pandemic policymaking.

We further assume that people’s informational needs are related to their normative expectations of journalists. As was stated above, the pandemic posed a highly uncertain situation to society at large and to people’s everyday lives. Research has established that individuals have different capacities to tolerate uncertainty or ambiguities and different ways of coping with it. While some prefer secure and stable knowledge that remains unchallenged by exceptions, others enjoy making up their minds based on conflicting information (Webster and Kruglanski, 1994). It is likely that news consumers’ informational needs differed due to such general individual differences and associated with different normative expectations of news coverage. We hypothesize the following:

**Hypothesis 2 (H2):** The more individuals sought information for orientation, the more they wanted journalists to deliver definite and unambiguous information about the pandemic.
**Hypothesis 3 (H3):** The more individuals sought information for construction, the more they wanted journalists to question policy measures and scientific advice.

### Epistemic beliefs

The public debate over managing the coronavirus pandemic involved highly specialized scientific knowledge which was reported intensively in the news. People have different concepts of the nature of scientific knowledge, that is, *epistemic beliefs* (Kardash and Scholes, 1996; Kienhues and Bromme, 2012). For example, some hold more sophisticated epistemic beliefs viewing scientific knowledge as tentative and theories as revisable, while others hold more naïve beliefs viewing scientific knowledge as certain and theories as invariable (Howell et al., 2020). We assume that a naïve view of scientific knowledge as certain and stable corresponds to a linear view of the relationship between science and policy that theoretical thinkers have dismissed as simplistic (Pielke, 2004; Sarewitz, 2004). Research has pointed in this direction finding that the more people subscribe to the belief that scientific knowledge is definite, the more they defer to scientific authority in societal decision making over science and technology (Howell et al., 2020). We hypothesize the following:

**Hypothesis 4 (H4):** The more individuals believe that scientific knowledge is certain and definite, the more they wanted scientists to dominate pandemic policymaking.

We also expect that people’s epistemic beliefs associated with their expectations of journalists. Research has shown that their epistemic beliefs relate to their understanding of scientific knowledge. For instance, the more individuals believe that scientific knowledge is certain and definite, the more they are prone to disregard existing uncertainties and ambiguities of scientific evidence to fit their views (Kardash and Scholes, 1996; Schommer, 1990). Assuming that their epistemic beliefs related to their expectations of news coverage in a similar way, we hypothesize the following:

**Hypothesis 5a (H5a):** The more individuals believe that scientific knowledge is certain and definite, the more they wanted journalists to deliver unambiguous, definite information about the pandemic.
**Hypothesis 5b (H5b):** The less individuals believe that scientific knowledge is certain and definite, the more they wanted journalists to question policy measures and scientific advice.

### Approval of public communication

As described above, mainstream news coverage of the early phase of the pandemic was criticized for its homogeneity, lack of critical questions, and concentration on few scientific sources. This suggests that the media met some members of the audience’s needs and expectations more than others. Based on research showing that news consumers’ expectations of the media predict their evaluations of media performance (e.g. Fawzi and Mothes, 2020; Lambe et al., 2004), we assume that, along with their informational needs and epistemic beliefs, citizens’ normative expectations of journalists, scientists, and policymakers predict their approval of the public pandemic crisis communication. For their informational needs, we hypothesize the following:

**Hypothesis 6a (H6a):** The more individuals sought information for orientation, the more they approved of the public pandemic crisis communication.
**Hypothesis 6b (H6b):** The more individuals sought information for construction, the less they approved of the public pandemic crisis communication.

For their epistemic beliefs, we hypothesize the following:

**Hypothesis 7 (H7):** The more individuals believe that scientific knowledge is certain and definite, the more they approved of the public pandemic crisis communication.

We further hypothesize that their expectations of the cooperation between scientists and policymakers relate to their approval of the public pandemic crisis communication, precisely that

**Hypothesis 8 (H8):** The more individuals wanted scientists to dominate pandemic policymaking, the more they approved of the public pandemic crisis communication.

Finally, we hypothesize that their expectations of journalists associate with their approval of the public communication, namely, that

**Hypothesis 9a (H9a):** The more individuals wanted journalists to deliver unambiguous, definite information, the more they approved of the public pandemic crisis communication.
**Hypothesis 9b (H9b):** The more individuals wanted journalists to question political measures and scientific advice, the less they approved of the public pandemic crisis communication.

## 4. Case study

The context of our study is the coronavirus crisis after infection numbers surged in Germany for the first time. After the new coronavirus SARS-CoV-2 (severe acute respiratory syndrome coronavirus 2) emerged in Wuhan, China, in December 2019, it rapidly culminated in a severe worldwide public-health crisis. In February 2020, a devastating outbreak in Italy attracted wide attention in Europe (Worobey et al., 2020). When case numbers were hovering in Germany at the beginning of March, the German government implemented various countermeasures (Robert Koch Institut, 2020b). On 16 March, public sites such as restaurants, churches, and movie theaters were closed, as were schools and universities. A week later, temporary contact restrictions for all citizens were enacted. Citizens were banned from meeting in public with more than three persons or more than two households. A minority of federal states, such as Bavaria, implemented additional lockdown measures. On 1 April, while cases were surging, the interventions were extended until 19 April. On 12 April, citizens were restricted to gather for Easter festivities. The measures proved effective at reducing transmission when the reproduction rate *R* dropped below 1.0 on 17 April (Robert Koch Institut, 2020a). On 20 April, the first coordinated exit strategies were employed, such as the opening of small shops. The period of severe restrictions for citizens ended on 30 April when playgrounds, churches, and museums opened up again.

When cases still surged, there was virtually no public disagreement over the measures taken to curb the disease (cf. Wormer, 2020). However, in mid-April, as infection rates were decreasing, public actors repeatedly demanded discussing exit strategies in public. Chancellor Angela Merkel criticized such calls pointing out that it was too early to relax the restrictions.^7^ But an increasing number of actors demanded public discussions of exit strategies and considerations of the negative side effects of the lockdown measures. On 7 April, the German Ethics Council released a statement explicitly encouraging public debates over exit strategies (Dabrock and Augsberg, 2020). On 13 April, the German National Academy of Science issued a statement calling attention to the negative side effects of the lockdown—for example, with regard to psychological, economic, social consequences (Leopoldina Nationale Akademie der Wissenschaft, 2020). This phase of a slowly emerging controversial public debate is the context of our study. Our survey was conducted between 9 and 14 April 2020 when the debate became more controversial. We assume that this provides an ideal setting to investigate what people with different informational needs and epistemic beliefs expected of journalists, scientists, and policymakers, and how they evaluated the public pandemic crisis communication.

## 5. Method

To test our hypotheses, we conducted a cross-sectional survey of 1513 participants of an online access panel administered by *Respondi*, a market research company located in Germany. The completion rate is 11.1% (American Association for Public Opinion Research, Response Rate 6, AAPOR RR6). The data were collected over Easter between 9 and 14 April 2020. Corresponding to the 2011 national census of the German population, 51% of the participants are female and 49% are male (Table 1). There is also correspondence with regard to age and respondents’ federal state of residence as well as near correspondence with regard to education. In our sample, there is a slight overweight of highly educated and a slight underweight of less educated people. All in all, with regard to key characteristics, our sample is approximately representative of the German population. Respondents were recruited via an online access panel, suggesting that active online users are overrepresented relative to the general population. However, as of 2020, Germany had an Internet penetration of 96% (Hölig & Hasebrink 2020). Furthermore, participation was facilitated for many participants because the data were collected over Easter during the first lockdown when many people spent their time at home rather than at work or meeting family. Based on this information, we assume that our sample is an approximate representation of the German population.

**Table 1.** Comparisons of demographic statistics in the sample and German population.

	Sample		Census data (2011)
	*n*	%	%
Age in years (*M* = 48.58, *SD* = 16.50)			
18–30	268	18	17
30–40	204	13	14
40–50	300	20	20
50–65	376	25	24
65+	365	24	25
Gender			
Female	774	51	51
Male	739	49	49
Education (*M* = 1.95, *SD* = 0.84)			
Low (1: lower secondary school diploma, or below)	576	38	43
Medium (2: intermediate secondary school diploma)	433	29	29
High (3: university qualifying school diploma, or above)	504	33	28

*N* = 1513 participants of an Online Access Panel representative of the German population with regard to age, gender, education, and federal state of residence. Surveyed between 9 and 14 April 2020. Census data retrieved from https://ergebnisse.zensus2011.de.

### Measures

#### Dependent variables

To measure respondents’ *wish that scientists dominate pandemic policymaking*, they were asked: “How should policymakers and medical scientists—e.g. virologists—cooperate in the coronavirus crisis?” On a 5-point scale, they gave their levels of agreement with four items expressing the view that medical scientists should dominate policymaking. The items and statistics of all variables are presented in Table 2. Descriptive results of the conceptual variables will be given in the “Results” section. A principal components analysis (PCA) reveals that the four items load on one factor. They were averaged to form a composite measure (Cronbach’s α = .797).

**Table 2.** Statistics of dependent and independent variables.

	*M*	*SD*
Expectation that science dominate policy (1 = *totally disagree*, 5 = *totally agree*; Cronbach’s α = .797)	4.07	0.68
“Politicians should definitively put medical scientists’ recommendations into practice.”	3.99	0.84
“Before politicians make decisions, they should have this confirmed by medical researchers.”	4.22	0.80
“Medical researchers should clearly tell policymakers what to do in a crisis.”	4.10	0.89
“Medical researchers should work to ensure that politicians implement their recommendations.”	3.99	0.92
Expectation that journalists deliver definite facts (1 = *totally disagree*, 5 = *totally agree; r_SB_* = .676)	4.42	0.68
“Journalists should largely base their reports on definite facts.”	4.51	0.75
“Journalists should primarily cite scientists who make certain claims.”	4.34	0.81
Expectation that journalists question science and policy (1 = *totally disagree*, 5 = *totally agree; r_SB_* = .745)	3.66	0.95
“Journalists should question the political measures taken to manage the crisis critically.”	3.80	1.02
“Journalists should question virologists’ recommendations on how society should manage the crisis critically.”	3.52	1.11
Approval of public pandemic crisis communication (1 = *totally disapprove*, 5 = *totally approve*; Cronbach’s α = .848)	3.35	0.95
Approval of news coverage	3.28	1.10
Approval of public communication of the government	3.25	1.13
Approval of public communication of scientists	3.53	1.04
Informational need for orientation (1 = *totally disagree*, 5 = *totally agree; r_SB_* = .74)	4.03	0.93
“I wanted definite information on the political measures taken in this crisis.”	4.16	0.97
“I wanted definite expert judgment that I could rely on.”	3.89	1.11
Informational need for construction (1 = *totally disagree*, 5 = *totally agree*)	3.67	1.21
“I was looking for different opinions to make my own judgment.”	3.67	1.21
Epistemic belief: Scientific knowledge is stable (1 = *totally disagree*, 5 = *totally agree*; Cronbach’s α = .712)	2.84	0.70
“Research in medicine has shown that there is one clear answer to most problems.”	3.05	1.00
“If physicians address themselves to the investigation of a question, they will find the correct answer to almost all questions.”	2.93	0.97
“If different physicians predict the course of a person’s disease, they almost always agree.”	2.82	0.96
“In medicine, there is not much to discuss—the facts speak for themselves.”	3.05	1.13
“Once a theory in medicine is proven true, it is valid forever.”	2.37	1.09
Demographics	See Table 1	
Vulnerability^a^		
Health-related vulnerability (Index, range = 0–10)	1.38	1.28
Economic vulnerability (Index, range = 0–9)	0.64	0.86
Media use (1 = *never*, 5 = *very often*)		
Use of public broadcasting	3.68	1.35
Use of private broadcasting	2.87	1.43
Use of online media (Cronbach’s α = .745)	2.61	0.97
Use of print media (*r_SB_* = .736)^b^	2.76	1.24
Use of social media (*r_SB_* = .746)	2.58	1.10

*N* = 1513 participants of an Online Access Panel representative of the German population with regard to age, gender, education, and federal state of residence. Surveyed between 9 and 14 April 2020.

a Vulnerability was measured based on 10 (health) and nine (economic) single-choice items. Single-choice items were combined in an averaged additive index.

b Spearman–Brown coefficient was used as reliability measure for two-item measures.

To measure people’s expectations of journalists, they were asked: “In the coronavirus crisis, one can expect different things of journalists. How much do you agree or disagree with the following claims?” They indicated their levels of agreement with several claims on a 5-point scale. Two expressed a wish that journalists deliver definite facts. Another two items expressed a wish that journalists question political measures and scientific advice. A PCA reveals that the four items load on two factors. We averaged the respective two items for each measure and calculated the Spearman–Brown coefficient as a measure of the reliability of two-item scales (Eisinga et al., 2013), with *r_SB_* = .676 for the expectation that journalists deliver definite facts and *r_SB_* = .745 for the expectation that journalists question scientific advice and policy.

To measure respondents’ *approval of public pandemic crisis communication*, they were asked to indicate how much they disapproved or approved of (a) news coverage, (b) public communication of members of the government, and (c) public communication of scientists on 5-point scales. A PCA shows that the three items load on one factor. We averaged respondents’ ratings (Cronbach’s α = .848).

#### Independent variables

Following Chew (1994), we measured respondents’ informational needs. They were asked for their reasons to turn to news on the pandemic in the past weeks. They rated their levels of agreement with four items on a 5-point scale. A PCA indicates that the four items load on one factor (Cronbach’s α = .799). However, based on theoretical considerations derived from Chew (1994), we sought to separate items relating to respondents’ informational needs for orientation and construction. For this reason, we ran a PCA set to extract two factors. Two of the items aimed at measuring citizens’ *informational need for orientation* loaded on one factor (“I wanted definite information on the political measures taken in this crisis,” “I wanted definite expert judgment that I could rely on”). One of the items aimed at measuring people’s *informational need for construction* loaded on the second factor (“I looked for different opinions to make up my own mind”). Another item loaded on both factors and hence was dropped from the analysis (“I wanted to comprehend how well the measures and recommendations were founded”). We thus base our analysis on an averaged two-item measure for citizens’ *informational need for orientation* (*r_SB_* = .74) and a one-item measure for *citizens’ informational need for construction*.

We measured respondents’ epistemic beliefs about medical knowledge following Kienhues and Bromme (2012). On a 5-point scale, respondents indicated their levels of agreement with five statements that capture a *static view of medical knowledge.* A PCA shows that the five items load on one factor. We averaged respondents’ ratings to form a composite measure (Cronbach’s α = .712).

#### Control variables

Besides demographics (gender, age, education), we controlled for respondents’ personal *economic* and *health-related vulnerability*. We asked: “Have you personally been affected by the coronavirus?” They were given 10 criteria (e.g. “I was infected,” “I belong to a risk group,” “I work in healthcare,” “Someone I know was or has been in quarantine”) and asked to indicate all that applied to them. We also asked: “Have you been affected by the coronavirus crisis economically?” They were given eight criteria plus an open option (e.g. “My working hours have been reduced,” “I had to lay off staff or reduce their hours,” “I have or expect financial problems”). To obtain a measure of their personal *health-related* and *economic vulnerability*, we summed up respondents’ data on each of these questions.

People’s trust in or attitudes to media news coverage has repeatedly been predicted by individuals’ media usage (e.g. Arlt, 2018; Jackob et al., 2019). Thus, we controlled for respondents’ use of information sources. We asked how they had stayed informed about the coronavirus crisis since the lockdown. On a 5-point scale, they indicated how often they had used 12 specific sources of information. Based on a PCA, we differentiated *online information consumption*, including use of news search engines, websites of executive authorities, websites of scientific institutions or federal research institutes, and alternative online news sites, *newspaper usage*, including use of regional and national newspapers, and *social media use*, including postings of friends and family as well as private messaging services. We further measured *public* and *private TV news use* by two single items.

## 6. Results

### Preliminary findings

Respondents were split on the perceived quality of public pandemic crisis communication by policymakers, media, and scientists—some were more, others were less content with it (Table 2). They were also split on the nature of scientific knowledge. On average, respondents tended to reject the belief that scientific knowledge was stable, but some still subscribed to it. Possibly partly due to the highly uncertain situation, respondents had a prevailing need for definite information and a desire for authoritative science. They largely expected that scientists should dominate pandemic policymaking and that journalists should deliver definite facts. When consuming the news, they largely sought definite information for orientation. By comparison, respondents’ wish that journalists question science and policymaking was less pronounced as was their interest in making up their own minds.

To test relationships between people’s normative expectations, approval of the public pandemic crisis communication, their informational needs, and epistemic beliefs, we computed ordinary least squares (OLS) regressions. In three separate models, we predicted audiences’ normative expectations of scientists, policymakers, and journalists. In an additional model, we predicted their approval of the public pandemic crisis communication. In each model, we first entered respondents’ demographics, information usage, and vulnerability as control variables. We then tested the effects of our conceptual variables over and beyond these control variables.

### Predicting people’s expectations

We expected that people’s informational needs and their epistemic beliefs associated with their normative expectations of journalists’ news coverage and of the cooperation between scientists and policymakers during the pandemic. The results largely confirm this. The more people sought definite information for orientation, the more they wanted medical scientists to dominate political decision making. By contrast, the more they sought information for constructing their own opinions, the less they wanted medical scientists to dominate policymaking (Table 3, first column). This confirms H1a and H1b.

**Table 3.** OLS regressions explaining people’s normative views of journalists, scientists, and policymakers by their informational needs and epistemic beliefs.

	People’s wish that . . .					
	. . . scientists dominate policy		. . . journalists report definite facts		. . . journalists provide criticism	
	*b* (*SE*)	β	*b* (*SE*)	β	*b* (*SE*)	β
Constant	2.678 (.134)		3.145 (.135)		3.000 (.194)	
Demographics and vulnerability						
Age	.003 (.001)	.065*	.002 (.001)	.050	.015 (.002)	.254***
Gender (1 = male, 2 = female)	.023 (.033)	.017	.049 (.033)	.036	−.101 (.048)	−.053*
Education	−.054 (.015)	−.091***	−.008 (.015)	−.014	−.032 (.022)	−.038
Health-related vulnerability	−.003 (.013)	−.006	.030 (.013)	.056*	−.054 (.019)	−.073**
Economic vulnerability	.010 (.020)	.012	−.012 (.020)	−.016	.050 (.029)	.045
Δ Adjusted *R*^2^	.021***		.020***		.059***	
Media use						
Use of public broadcasting	.044 (.014)	.087**	.032 (.014)	.064*	−.067 (.020)	−.095**
Use of private broadcasting	−.007 (.012)	−.015	−.005 (.012)	−.010	−.002 (.017)	−.003
Use of online media	.021 (.020)	.031	−.030 (.020)	−.043	−.019 (.029)	−.020
Use of print media	.008 (.015)	.014	−.001 (.015)	−.003	.028 (.022)	.036
Use of social media	.021 (.017)	.034	.016 (.017)	.027	.070 (.024)	.082**
Δ Adjusted *R*^2^	.043***		.020***		.016**	
Conceptual variables						
Epistemic belief: Scientific knowledge is stable	.169 (.024)	.173***	.044 (.024)	.045	−.017 (.034)	−.012
Informational need: orientation	.218 (.021)	.296***	.259 (.021)	.355***	−.029 (.030)	−.029
Informational need: construction	−.051 (.015)	−.090**	−.034 (.015)	−.060*	.133 (.022)	.169***
Δ Adjusted *R*^2^	.102***		.097***		.021***	
Adjusted *R*^2^	.166***		.137***		.096***	

VIF: variance inflation factor.

*N* = 1513; VIF < 1.6, Durbin–Watson [1.98; 2.00].

* *p* < .05, **p < .01, ***p < .001.

Respondents’ informational needs also relate to their expectations of journalists, as expected (Table 3, second and third columns). The more people sought news for orientation during the lockdown, the more they wanted journalists to deliver definite information. By contrast, the more people sought news for construction, the more they wanted journalists to question political measures and scientists’ recommendations. This confirms H2 and H3. In addition, there was a negative relationship between respondents’ informational need for construction and their wish that journalists deliver definite information. The more people sought information for construction, the less they wanted journalists to deliver definite, unambiguous news. Albeit small, this association lends further support to the assumption that news users seeking information for construction expect journalists to do justice to ambiguities.

Beyond their informational needs, we expected that people’s epistemic beliefs predict their normative expectations of journalists, scientists, and policymakers. This is only partly confirmed. We assumed that people’s belief that scientific knowledge is certain, unambiguous, and invariable relates to their normative expectations of the cooperation between scientists and policymakers, hypothesizing that the more individuals believe that scientific knowledge is certain and definite, the more they wanted scientists to dominate policymaking in the pandemic. Our regression analysis largely confirms this. The more respondents believed that scientific knowledge is certain, the more they wanted medical scientists to dominate policymaking. This confirms H4.

We further assumed that the more people believe that scientific knowledge is certain and invariable, the more they wanted journalists to report in a definite, unambiguous way. This relationship is only approaching statistical significance and it is so weak that it hardly bears practical relevance. We also assumed that the more people subscribed to a view that scientific knowledge is certain, the less they wanted journalists to question science and policy. This relationship is clearly disconfirmed. People’s epistemic belief that scientific knowledge is certain does not relate to their wish that journalists question policy and scientific recommendations. Thus, H5a and H5b are not supported.

### Predicting people’s approval of public communication

We assumed that people’s informational needs and epistemic beliefs associate with their approval of the public pandemic crisis communication by the media, scientists, and policymakers. The regression analysis confirms this (Table 4). The more respondents sought definite and unambiguous information for orientation in the pandemic, the more they approved of the public communication. They approved less of it, by contrast, the more they sought information for constructing their own opinions. This confirms H6a and H6b. As expected, respondents’ approval also depended on their epistemic beliefs. People’s approval of the public pandemic crisis communication was higher, the more they believed that medical scientific knowledge is certain, definite, and invariable. This confirms H7.

**Table 4.** OLS regression explaining people’s approval of the public pandemic crisis communication by their normative views, informational needs, and epistemic beliefs.

	*b*	*SE*	β	*p*
Constant	.934	.219		<.001
Demographics and vulnerability				
Age	.001	.001	.025	.332
Gender (1 = male, 2 = female)	.077	.043	.040	.072
Education	.062	.019	.074	<.001
Health-related vulnerability	−.003	.017	−.004	.843
Economic vulnerability	−.052	.026	−.047	.045
Δ Adjusted *R*^2^	.029***			
Media use				
Use of public broadcasting	.155	.018	.218	<.001
Use of private broadcasting	.020	.015	.030	.191
Use of online media	.036	.026	.036	.175
Use of print media	.052	.019	.067	.008
Use of social media	−.073	.022	−.084	<.001
Δ Adjusted *R*^2^	.123***			
Conceptual variables				
Epistemic belief: Scientific knowledge is stable	.255	.031	.188	<.001
Informational need for orientation	.206	.029	.200	<.001
Informational need for construction	−.066	.020	−.083	.001
Expectation that. . .				
. . . science dominate policy	.101	.035	.072	.004
. . . journalists deliver definite facts	.071	.035	.050	.045
. . . journalists question science and policy	−.190	.023	−.189	<.001
Δ Adjusted *R*^2^	.132***			
Adjusted *R*^2^	.284***			

VIF: variance inflation factor.

*N* = 1513; VIF < 1.7; Durbin–Watson = 2.02.

* *p* < .05, **p < .01, ***p < .001.

We further anticipated that people’s normative expectations of journalists, scientists, and policymakers predicted their approval of the public pandemic crisis communication. The regression analysis confirms this though some relationships are rather weak. People who wanted scientists to dominate policymaking had a higher approval of the public pandemic crisis communication, but this association was weak. This confirms H8. In addition, people’s normative views of journalists predicted their evaluation of public communication. Again, with a weak association, individuals who wanted journalists to deliver definite information had a higher approval of the public pandemic crisis communication. By contrast, with a moderate effect size, individuals who wanted journalists to question science and policy had a lower approval. This is in line with H9a and H9b.

To sum up, in the early phase of the pandemic, Germans had different expectations of journalists, scientists, and policymakers depending on their informational needs and views of the nature of scientific knowledge. People seeking definite information for orientation in the crisis and people who embraced a view of scientific knowledge as stable and certain had similar expectations and perceptions. They were more content with the public pandemic crisis communication than others and tended to expect scientists to dominate pandemic policymaking. Furthermore, people with a strong informational need for orientation had a stronger expectation that journalists deliver definite information. By contrast, people seeking contradictory information to develop their own opinions were less content with the public pandemic crisis communication. They tended to reject the idea that scientists dominate pandemic policymaking and to expect that journalists criticize policymaking and scientific advice. People’s expectations, in turn, partly accounted for their approval of the public pandemic crisis communication. People who wanted scientists to dominate policymaking and journalists to deliver definitive facts tended to be more content whereas those who expected journalists to question policy and scientific advice tended to be less content.

## 7. Discussion

The coronavirus pandemic has repeatedly been termed one of the greatest challenges for societies in the industrialized world since the end of World War II. Touching upon highly specialized, complex, and fragmented scientific knowledge, the crisis was difficult to communicate in public. This study shows that different members of the news audience have different informational needs and thus prefer different communication styles. The results show that, in the early phase of the pandemic, policymakers, scientists, and journalists largely met the needs of people who sought certainty and definite information. However, people who sought to make up their own minds were less content. In tendency, these people desired journalists, scientists, and policymakers to open up debate over policymaking. While political decision makers might be tempted to suppress a controversial public debate in an imminent existential crisis for the sake of efficient action, social researchers should investigate whether this might backfire in the long run by making those with a desire for debate increasingly distrustful.

Against the backdrop of theories of democracy and science, our data suggest that people’s approval of the public communication was partly based on unattainable premises. For example, people were more content with the public pandemic crisis communication, the more they naively believed that scientific knowledge was stable and certain. They were also more content, the more they wanted journalists to deliver definite information—something that could hardly be accomplished in the highly uncertain situation of the pandemic. Moreover, their approval of the public pandemic crisis communication grew with their wish that scientists dominate policy—a view that may, at least in part, be problematic from the perspective of democratic theory.

As previous research (e.g. Hibbing and Theiss-Morse, 2003; Howell et al., 2020), our results hint at a weak spot in democratic decision making on issues involving scientific knowledge. Confronted with the coronavirus pandemic, many Germans appeared to compensate the lingering uncertainty by their preference for secured facts and expert authority in political decision making. Citizens’ resistance to ambiguity and their proneness to “defer to scientific authority” (Brossard and Nisbet, 2007) would be especially problematic, if it collided with scientists or political actors using select pieces of scientific knowledge as seemingly definite justifications for particular lines of political action. Thereby, scientists and political actors would preclude public debate instead of stimulating debate over policy options, values, and priorities (Pielke, 2004).

In the face of such challenges, it appears desirable to seize measures to enhance people’s understandings of the nature of scientific knowledge and of the relationship between science and policy. This seems even more important considering that societies face several major political problems touching upon scientific knowledge, for example, relating to climate change and sustainable food production. It seems desirable that policymakers and scientists involved in such debates make the boundaries between science and policymaking transparent—as scientists in the pandemic in Germany persistently did. As illustrated at the beginning of this article in an anecdote, virologists and epidemiologists repeatedly pointed out the practical implications of their research—for example, with regard to the openings or closures of schools—simultaneously stressing that they could not decide on societal priorities. In this regard, scientists’ public communication during the pandemic appears to be different from the public communication by scientists in other policy relevant fields (Post, 2016; Post and Ramirez, 2018).

As was stated, people’s wish for definite scientific facts might partly be naïve. However, there might also be a downside to people’s desire to make up their own minds on issues involving complex scientific knowledge. There is reason to question laypeople’s ability to discard relevant information on a highly complex and specific scientific issue. Research has shown that laypeople tend to overestimate their capacities to understand scientific knowledge (Fernbach et al., 2019; Schäfer, 2020; Scharrer et al., 2017). There might also be a downside to people’s rejection of the view that scientific knowledge is certain. In extreme cases, it might amount to a radical relativist position purporting that scientific knowledge totally depends on subjective viewpoints. It appears relevant to investigate how people’s objective and self-rated knowledge as well as their epistemic beliefs relate to their trust in science (cf. Howell et al., 2020).

As any study, this study has limitations. One is its context dependence. The early phase of the coronavirus pandemic provided an ideal case to investigate people’s informational needs and views of the relationships between science, policy, and the public under uncertainty. Yet, it also provided an extreme state that differed markedly from other situations, for example, with regard to people’s news consumption, their personal involvement, or social cohesion. While it is conceivable that some of the relationships found in this study are applicable to other cases, it is desirable to test the relationships in other contexts—for example, in the debate over climate change.

Second, our regression models imply causal relationships that we cannot test based on a correlational study. Yet theoretically, it seems plausible that people’s general informational needs precede their specific expectations of journalists, scientists, and policymakers in the crisis. Similarly, it seems plausible that their expectations precede their evaluations of the public pandemic crisis communication. Nevertheless, it would be desirable to conduct longitudinal studies in the future to test how people’s informational needs, normative expectations, and media evaluations interact and change over time.

Third, some of the scales used in our study should be improved in future research. Some of our measures—for example, on people’s informational needs and expectations of journalists—were based on one- or two-item measures. It seems desirable to develop scales based on more items to increase reliability. What is more, our scale measuring citizens’ conceptualizations of the relationship between science and policymaking should also be tested and validated in other contexts.

All in all, we hope to open up a range of questions for communication research that are relevant in the years to come. At the center of this research lies the question as to how science and its relationship to policymaking is conceived among the citizenry in media democracies. In view of future public debates over the environment, science, and technology, it would be worthwhile to follow up on some of the questions our study raises. To prepare for the societal challenges ahead, it seems essential to deepen our knowledge of how modern societies utilize scientific knowledge for political decision making while engaging citizens to discuss options, values, and priorities.

## Notes

1. Anne Will (2020).

2. Laschet (Christian Democrats, CDU): Mir sagen nicht Virologen, was ich zu entscheiden habe (2020). Deutschlandfunk [German public radio], 9 April.

3. As reported in several media such as Xanke (2020).

4. Translated by the authors from the German original: “wo der denkende Forscher aufhört und der wollende Mensch anfängt zu sprechen.”

5. ZAPP (2020).

6. For example, in the first months of the pandemic, some pointed out that journalists missed out on criticizing that the government health research agency, the Robert Koch Institute, only reported absolute numbers of infection cases without reporting nor monitoring the numbers of SARS-CoV-2 (severe acute respiratory syndrome coronavirus 2) tests conducted (Meier and Wyss, 2020; Ruß-Mohl, 2020).

7. As reported by several media such as Stöhr (2020).
